# Design of a Tripod-Shaped Radiator Patch Antenna for Ultra-Wideband Direction Finding

**DOI:** 10.3390/s23229157

**Published:** 2023-11-13

**Authors:** Sangwoon Youn, Sungsik Ohm, Byung-Jun Jang, Hosung Choo

**Affiliations:** 1 Department of Electronic and Electrical Engineering, Hongik University, Seoul 04066, Republic of Korea; tirano88@mail.hongik.ac.kr; 2 Tactical Communication System Team, Hanwha Systems, Seongnam 13524, Republic of Korea; sungsik.ohm@hanwha.com; 3 Department of Electrical Engineering, Kookmin University, Seoul 04066, Republic of Korea; bjjang@kookmin.ac.kr

**Keywords:** array system miniaturization, single radiator multiple ports, UWB direction finding, channel impulse response

## Abstract

As UWB technology develops and devices become smaller, miniaturization techniques for an array antenna system are required. In addition, more in-depth research is needed for UWB direction-finding techniques using channel impulse response (CIR) data. This paper proposes an ultra-wideband (UWB) antenna using a single-radiator multiple-port (SRMP) design for the direction-finding systems of smart devices. The proposed SRMP antenna was designed using a single tripod-shaped patch that can replace the array system. The tripod-shaped radiator was optimized using the edge shape design function to improve its broadband and mutual coupling characteristics. For performance verification, the proposed antenna was fabricated, and the reflection coefficient, mutual coupling, and radiation patterns were measured in a fully anechoic chamber. The proposed antenna has an operating frequency band of 6.1 GHz (from 5.8 GHz to 11.9 GHz) for port 1 and a measured mutual coupling of −14.8 dB at 8 GHz. The SRMP antenna has measured maximum gains of 3.5 dBi for port 1 and 2.9 dBi for port 2. To examine the direction-finding performance, the fabricated antenna was connected to a circuit module with a DW3000 chip, which is widely employed in commercial mobile UWB systems. The direction of arrival (DoA) results using the measured CIR data show root-mean-square (RMS) errors of 1.57° and 4.58° at distances of 30 cm and 60 cm.

## 1. Introduction

Recently, there has been a growing interest in ultra-wideband (UWB) localization technology at a short distance for application in indoor real-time location systems, automotive radar systems, virtual reality, and augmented reality [1,2,3,4,5]. UWB localization technology has the advantage of obtaining a high resolution within an error range of 10 cm due to a broad bandwidth of more than 500 MHz. There are some studies about accurate localization solutions using gradient clock synchronization algorithms [6], autonomously powered systems [7,8], and propagation characteristics for non-line-of-sight (NLoS) signals [9,10]. Therefore, there is a trend of replacing the existing Wi-Fi and Bluetooth technology, previously used for indoor real-time location systems, with UWB technology, which can be implemented with high accuracy and low cost. For example, localization performance was improved with the method of distinguishing LoS/NLoS propagation [11], and a low-cost localization system was reported with a simple signal processing architecture [12,13,14]. UWB localization operates by converging distance information through time of flight (ToF) and direction information through angle of arrival (AoA) to obtain the location information of a target device or tag. Localization performances using only the ToF with several antenna modules are investigated in [15,16], and localization performances using both ToF and AoA with array antenna systems are analyzed in [17,18]. To obtain a direction-finding result through AoA, an array antenna system with more than three antenna elements is required [19,20,21,22]. Typically, an array spacing of about half wavelength is used to measure the phase difference of received signals through each antenna, and this spacing requires sufficient mounting space for array antennas. As technology develops and devices to which UWB technology is applied become smaller, miniaturization techniques for an array antenna system are extensively studied. Therefore, various studies on the miniaturization of individual antenna elements have been conducted for compact array antenna systems [23,24,25]. However, studies such as array antenna design using a slot aperture (5.1 λ at the center frequency of 64 GHz) and a stair-shaped radiator (1.05 λ at the lowest frequency of 3 GHz) have limitations in that the spacing between the array elements is still required even though the size of each element has been reduced [26,27]. As the array spacing narrows, the mutual coupling characteristics between array elements deteriorate. To improve isolation characteristics, a ladder-shaped metal via or a DGS structure is applied, but this requires additional structures. In addition, more in-depth research is needed on UWB direction-finding techniques, particularly for small array antenna systems using channel impulse response (CIR) data.

In this paper, we propose a single-radiator multiple-port (SRMP) antenna for UWB direction-finding applications which is more improved with a patch-type design based on the previous publication [28]. The proposed antenna is designed considering the limited mounting space so that it can replace an array system using multiple radiators. This patch-type SRMP antenna is optimized using the edge shape design function of the tripod-shaped radiator. The edge shape design function is employed to achieve higher gain within a smaller aperture size compared to the previously published SRMP antenna [29] while improving its broadband and mutual coupling characteristics. The three ports are then arranged on the edge of the tripod-shaped radiator. For performance verification, the proposed SRMP antenna was fabricated, and the reflection coefficient, mutual coupling, and radiation patterns were measured in a fully anechoic chamber. To examine the direction-finding performance when it is mounted on smart devices, the fabricated antenna was connected to a circuit module with Qorvo’s DW3000 chip for achieving UWB CIR data, which has not been studied in previous research. To adjust the operating frequency and pulse interval of the transmission signal, a Nordic board nRF52840 DK was connected to control the DW3000. Then, the proposed SRMP antenna was connected to the receiving module, and the direction of arrival (DoA) estimation results according to the incident angle from −30° to 30° were measured. The results demonstrate that the proposed SRMP antenna using a tripod-shaped radiator is suitable for UWB direction-finding system usage in smart devices.

## 2. Design of the Tripod-Shaped SRMP Antenna

### 2.1. Proposed SRMP Antenna Design

**Figure 1:**
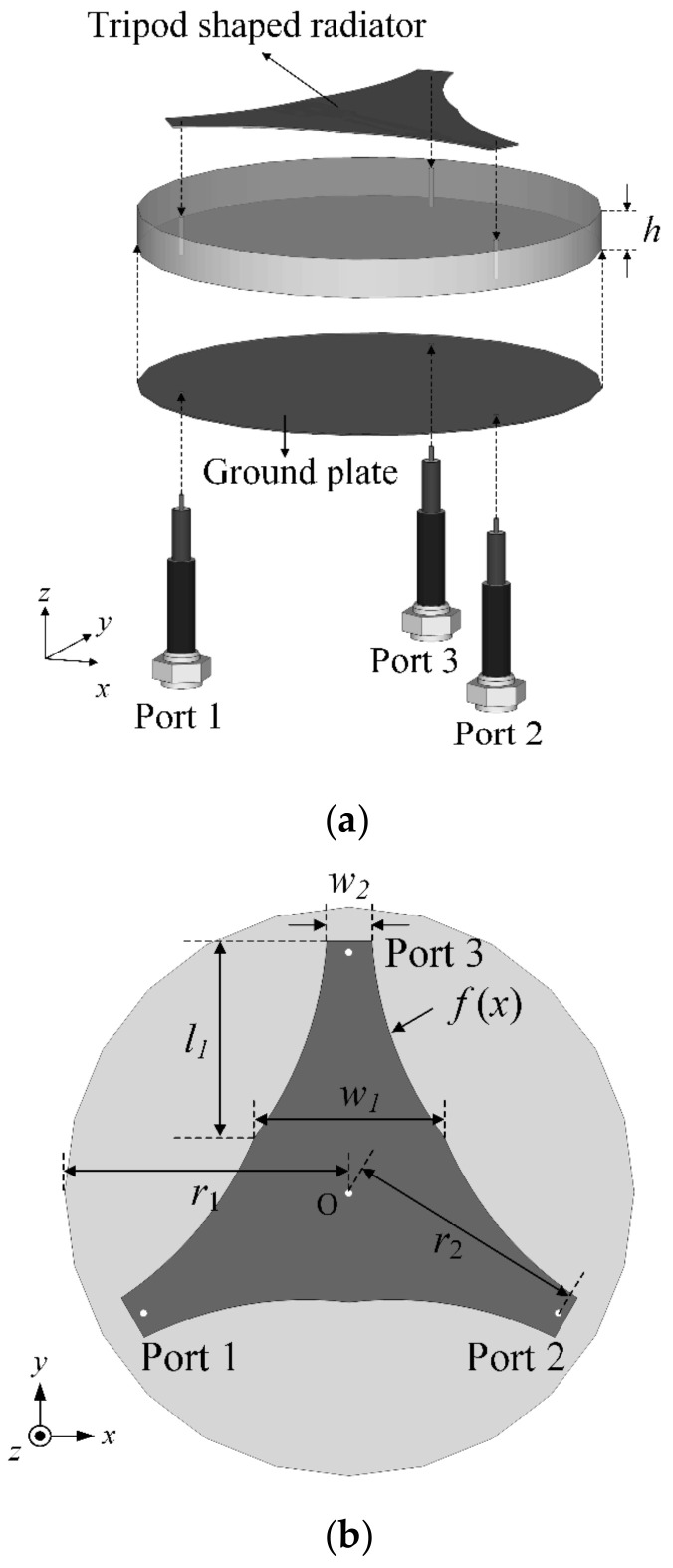
Geometry of the proposed tripod-shaped patch antenna: (**a**) isometric view; (**b**) top view.

Figure 1a shows the geometry of the proposed UWB direction-finding antenna, which uses a tripod-shaped radiator with three feeding points, in isometric view. Considering the limited mounting space of smart devices, the proposed SRMP antenna is designed using a single patch that can replace the array antenna system with decreased mounting space. The tripod-shaped patch radiator has widths of *w*_1_ and *w*_2_ and a length of *l*_1_, as shown in Figure 1b. The radiator is printed on an FR-4 substrate (*ε_r_* = 4.3, tan *δ* = 0.018) with a radius of *r_1_* and a height of *h*. The edge curve of the tripod-shaped radiator is designed with the function *f*(*x*) to obtain broadband characteristics while maintaining low mutual coupling. The design function *f*(*x*) is the quadratic polynomial expressed in Equations (1) and (2), where *k* is determined by *w*_1_, *w*_2_, and *l*_1_. The three ports are arranged at the edge of the tripod-shaped radiator at a distance of *r*_2_ from the center. The proposed SRMP antenna was modeled and simulated using the CST Studio Suite EM simulator [30]. A genetic algorithm (GA) with 20 generations was used for detailed design optimization, and the optimized parameters were then obtained in the 20th generation. The optimized design parameters are listed in Table 1.

(1) $$f\left(x\right)=k\times {\left(x-\frac{w_{1}}{2}\right)}^{2}+\frac{w_{1}}{2\sqrt{3}},$$

(2) $$\frac{w_{2}}{2}<x<\frac{w_{1}}{2\sqrt{3}},k={\left(\frac{2}{w_{2}-w_{1}}\right)}^{2}\times l_{1}.$$

### 2.2. Fabrication and Measurement Results

**Figure 2:**
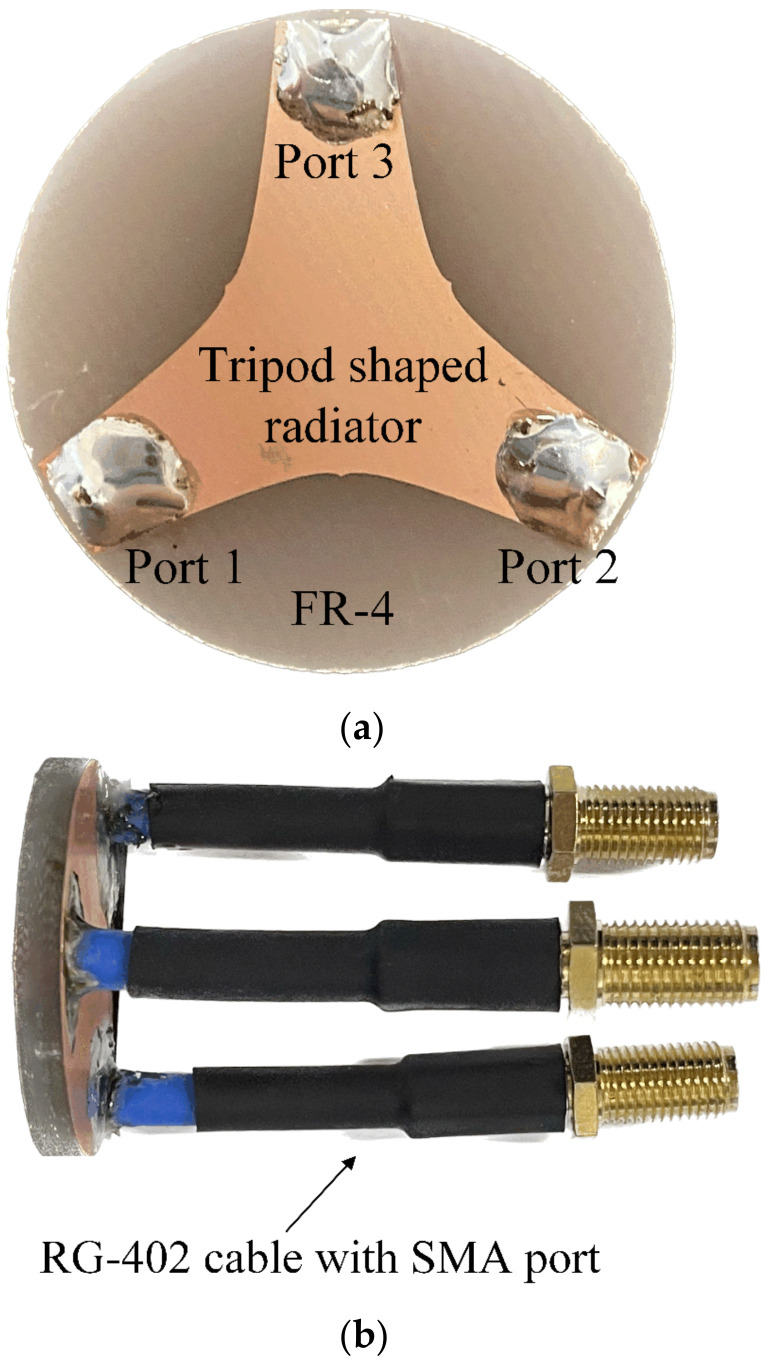
Photographs of the fabricated SRMP antenna: (**a**) top view; (**b**) side view.

**Figure 3:**
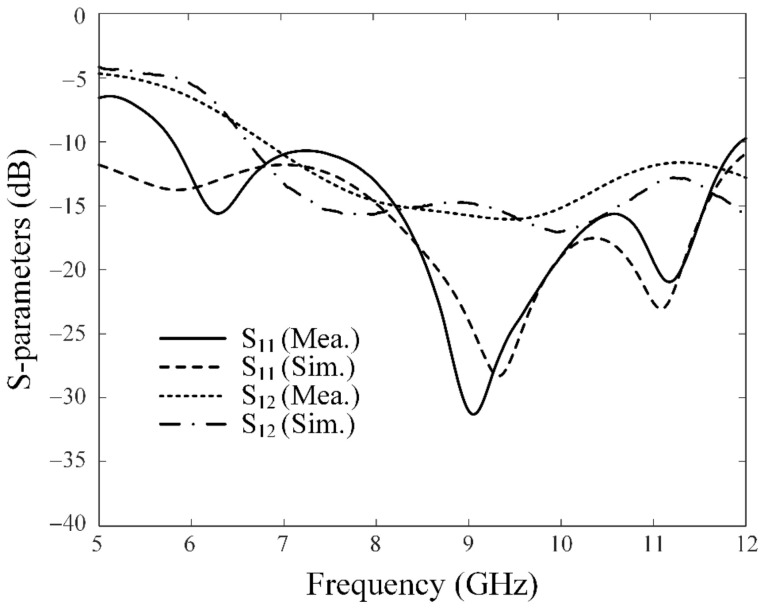
Simulated and measured S-parameters of the proposed SRMP antenna.

**Figure 4:**
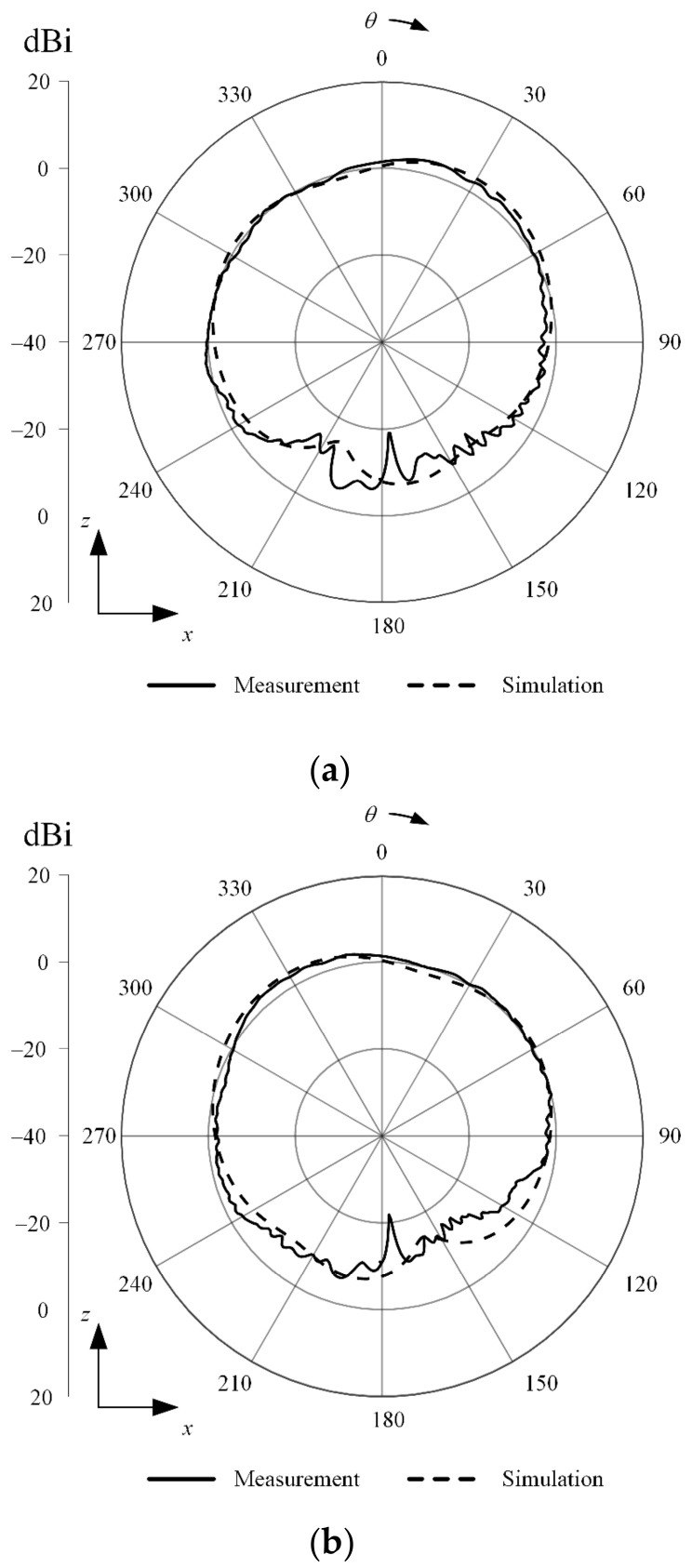
Simulated and measured radiation patterns of the proposed antenna in the *zx*-plane: (**a**) radiation patterns for port 1; (**b**) radiation patterns for port 2.

**Figure 5:**
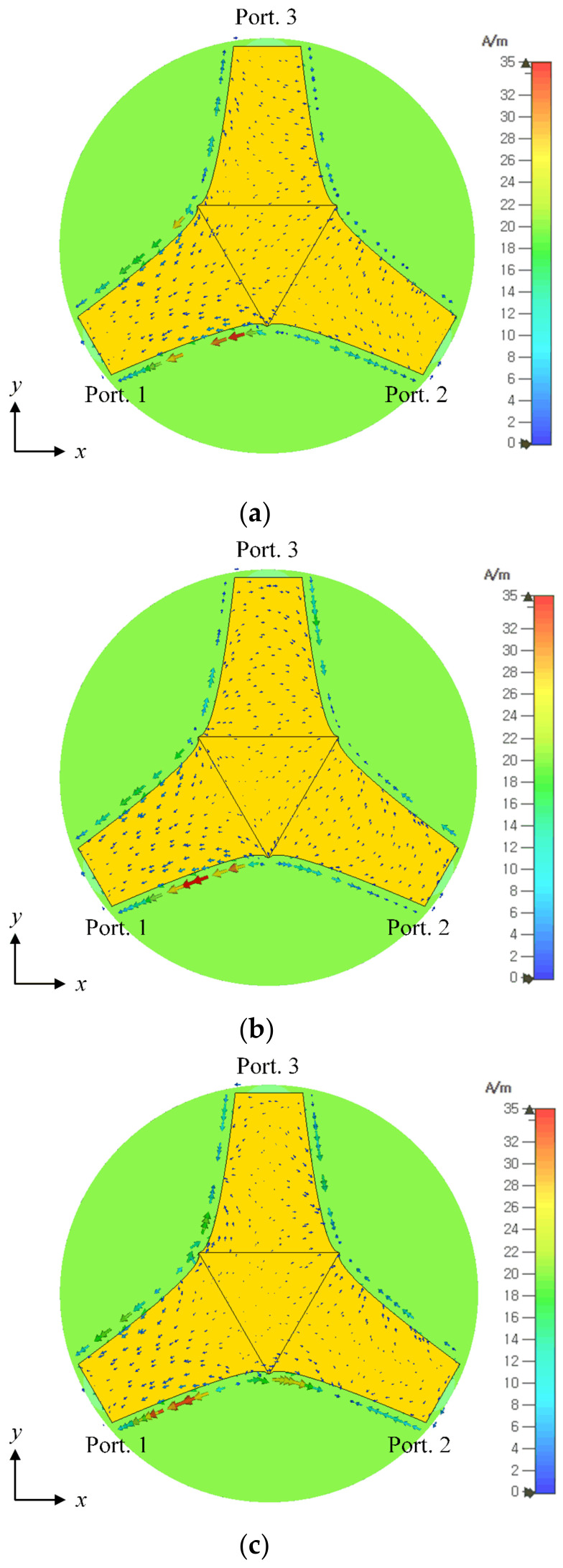
Simulated current distributions for port 1 in terms of frequency: (**a**) 7 GHz; (**b**) 8 GHz; (**c**) 9 GHz.

Figure 2a shows a photograph of the fabricated SRMP antenna printed on the circular FR-4 substrate. The proposed antenna is fed by three RG-402 cables that have low loss characteristics over the X-band, as shown in Figure 2b. The simulated and measured S-parameters of the SRMP antenna are shown in Figure 3. The operating frequency band observed at port 1 has a measured bandwidth of 6.1 GHz (from 5.8 GHz to 11.9 GHz), which is enhanced by 3 GHz compared to the previous publication. The simulation of −14.9 dB agrees well with the measurement of −13.3 dB at 8 GHz. The simulated total active reflection coefficient (TARC) of −5.2 dB is observed at 8 GHz for port 1, which is similar to the results for other ports due to the symmetrical geometry of the antenna. The measured and simulated mutual couplings of −14.8 dB and −15.5 dB are obtained between ports 1 and 2 at 8 GHz, respectively. Figure 4 presents the measured and simulated 2D radiation patterns of the proposed antenna in the *zx*-plane for ports 1 and 2. The maximum gain of 3.5 dBi is observed at *θ* = 30° for port 1 and 2.9 dBi at *θ* = −28° for port 2, within the small aperture size of 30.1 mm. The radiation efficiency and the envelope correlation coefficient (ECC) were also investigated for the proposed antenna. The simulated radiation efficiency of 75.9% is observed at 8 GHz for each port, and the average ECC of 0.134 is obtained between port 1 and port 2 at the target frequency range. Figure 5 shows the simulated current distributions for port 1 in terms of frequency under the condition that 1 W of input power is applied.

### 2.3. Optimization Using Design Parameters

**Figure 6:**
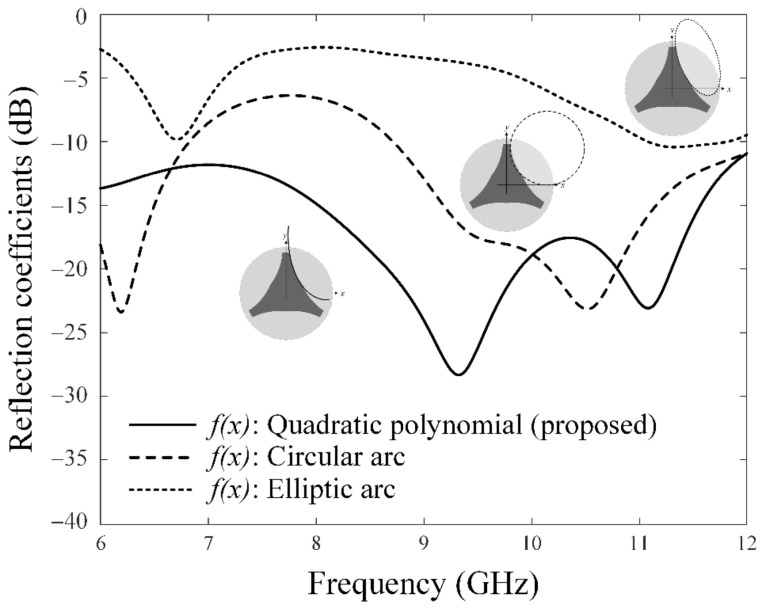
Reflection coefficients depending on design function *f*(*x*).

**Figure 7:**
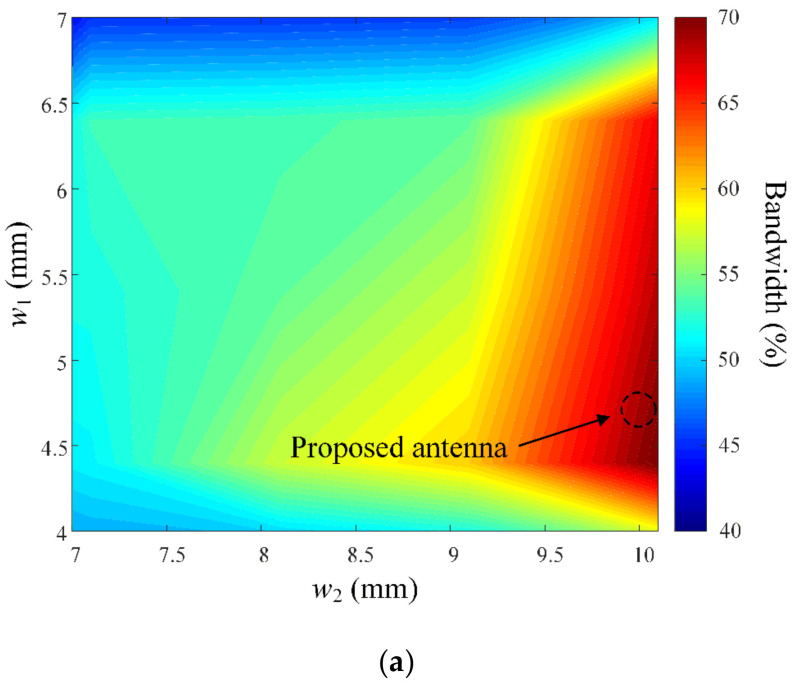

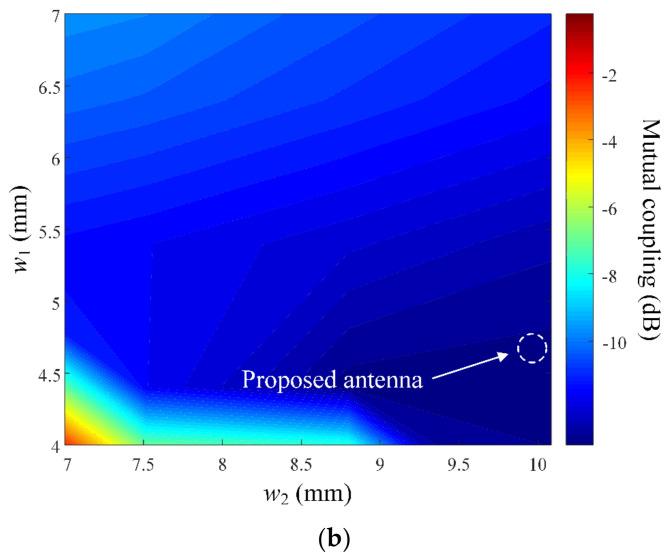
Optimization of the tripod structure for bandwidth and mutual coupling: (**a**) bandwidth according to *w*_1_ and *w*_2_; (**b**) mutual coupling according to *w*_1_ and *w*_2_.

Figure 6 shows the reflection coefficients depending on the design function *f*(*x*) for the upper right corner of the tripod-shaped radiator. The bandwidth characteristics were examined by varying the design function *f*(*x*) into an elliptic arc, a circular arc, and the proposed quadratic polynomial as shown in three inset figures. The tripod radiator with *f*(*x*) of the elliptic arc (dotted line) does not operate in the target frequency band. The result with *f*(*x*) of the circular arc (dashed line) has a broad bandwidth in the high-frequency band over 8.8 GHz. However, it does not achieve a reflection coefficient of less than −10 dB at 8 GHz. By contrast, the tripod-shaped radiator with *f*(*x*) of the quadratic polynomial (solid line), shown through Equations (1) and (2), can operate in the entire frequency band from 6 GHz to 12 GHz. Therefore, the essential design parameters of widths *w_1_* and *w_2_* were investigated to optimize the broadband characteristics with low mutual couplings using a single radiator without an additional component. The variations in the bandwidth according to *w_1_* (from 4 mm to 7 mm) and *w_2_* (from 7 mm to 10 mm) are presented, as shown in Figure 7a. The proposed antenna has a bandwidth of more than 65% when *w_2_* is wider than 10 mm. Figure 7b illustrates the mutual couplings at the target frequency of 8 GHz, with variations in widths *w_1_* and *w_2_*. When *w_1_* is 4.87 mm, the results of the mutual coupling are less than −12 dB. As a result, the *f*(*x*) of the tripod structure is optimized with *w_1_* of 10.1 mm and *w_2_* of 4.87 mm. The resulting mutual couplings are less than −14.8 dB, while maintaining a fractional bandwidth of 76.3%. The optimized design parameters of the proposed antenna which are improved with a tripod-shaped radiator using design function *f*(*x*) are compared to previous publications as listed in Table 2.

## 3. DoA Estimation Using the CIR Data

**Figure 8:**
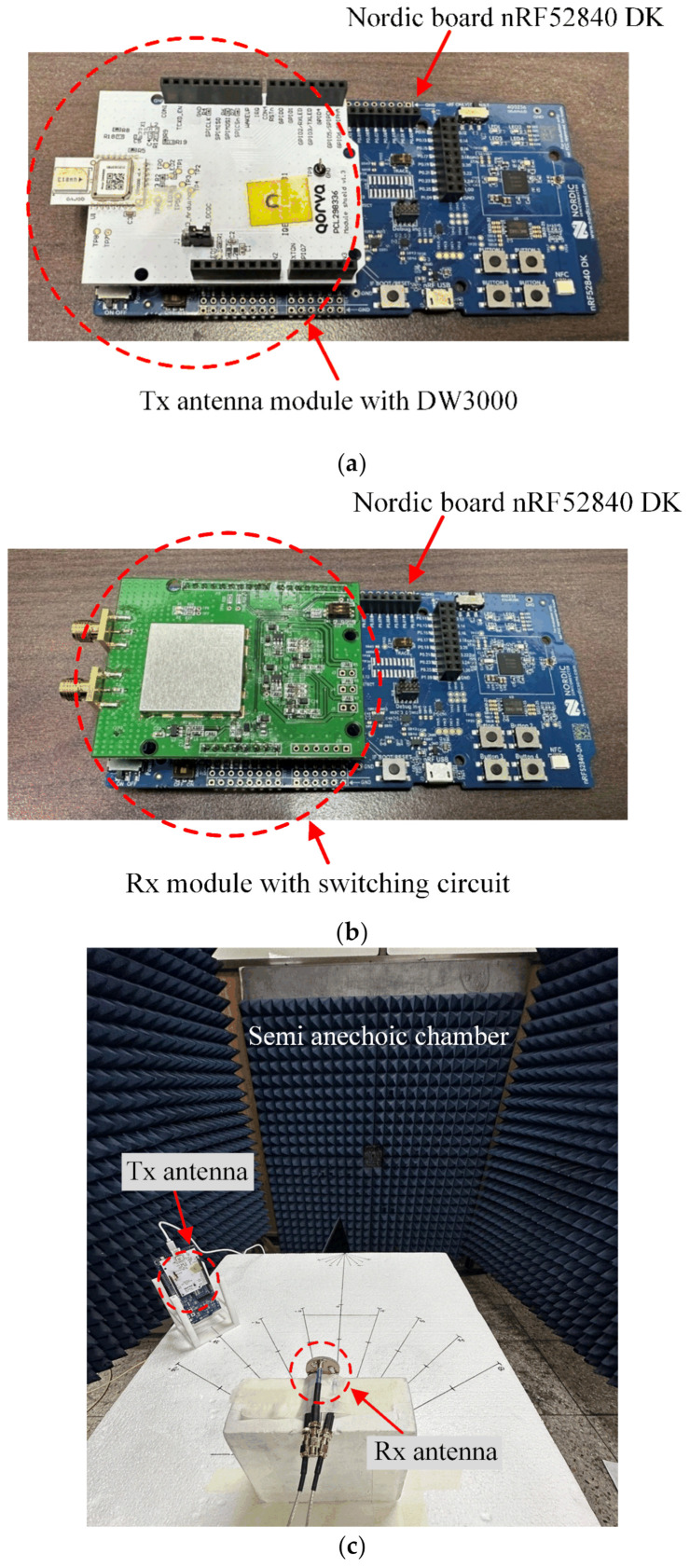
Measurement setup: (**a**) Tx board; (**b**) Rx board; (**c**) measurement setup for direction finding.

To investigate the DoA estimation performance of the proposed antenna using the CIR data, the DW3000 chip, which is widely employed in commercial UWB direction-finding systems, was used. As the transmitting antenna module, a test board with a monopole chip antenna and the DW3000 provided by Qorvo was used, as shown in Figure 8a. To adjust the operating frequency and pulse interval of the transmission signal, a Nordic board nRF52840 DK was connected to control the DW3000 chip. The application programming interface (API) that can control the DW3000 is accessed through the SEGGER Embedded Studio program on the computer. The transmit module was set to transmit pulses at the frequency of UWB channel 9 (from 7.737 GHz to 8.2368 GHz) with 500 μsec intervals. For the receiving antenna module, the DW3000 circuit board, including the SMA ports, was designed so the proposed antenna can be connected, as shown in Figure 8b. The circuit board was connected to the Nordic board in the same way as the transmission antenna module and collected the signal data received through the two SMA ports. Figure 8c shows the measurement setup for estimating the direction-finding performance of the proposed antenna in a semi-anechoic chamber. The proposed SRMP antenna connected to the receiving module was fixed on the Styrofoam jig. The transmit module moved at 5° intervals at a distance of 30 cm and 60 cm around the receiving module.

**Figure 9:**
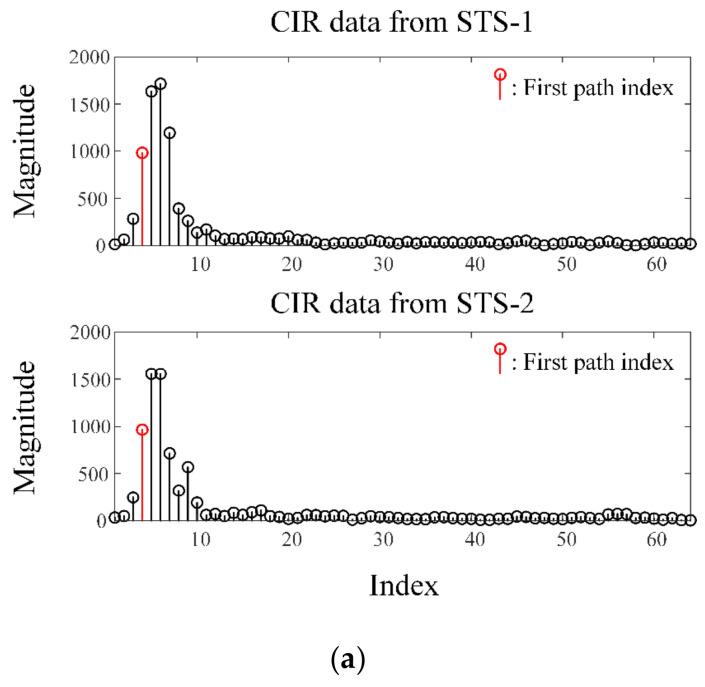

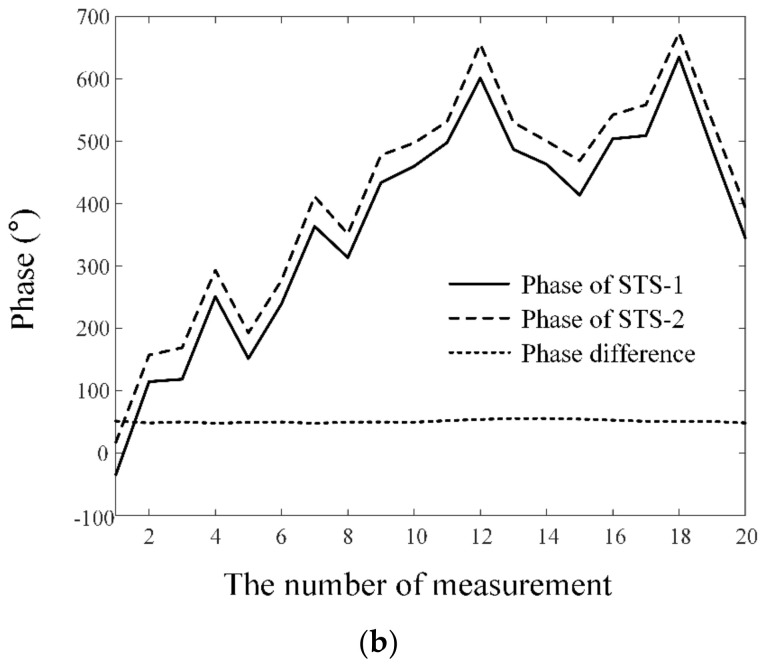
Comparisons of the CIR data and phase information of each STS signal: (**a**) CIR data according to STS-1 and STS-2; (**b**) phase information according to the number of measurements.

**Figure 10:**
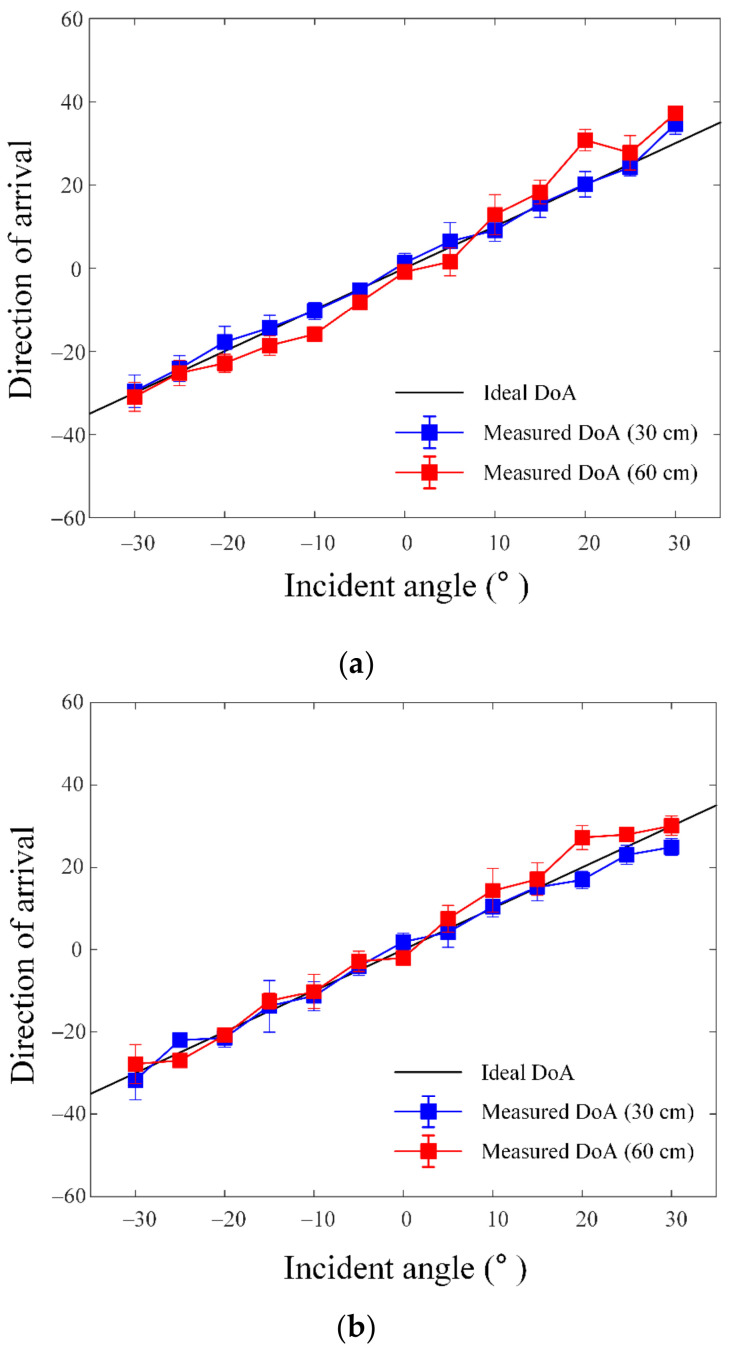
Measured DoA estimation result according to the incident angle: (**a**) DoA estimation result for *ϕ* = 0°; (**b**) DoA estimation result for *ϕ* = 60°.

Figure 9a shows the examples of the measured CIR data from ports 1 and 2 using the proposed antenna. We utilized the CIR data of the scrambled timestamp sequence (STS) field, which is added to provide secure ranging between transmitters and receivers based on IEEE Std 802.15.4z [31]. The CIR data were measured 20 times at 500 μsec intervals through the receiving module and represented as 64 indices, including the first path index (FPI). The FPI is the first CIR index that exceeds the predefined threshold. In the line-of-sight environment, a single pulse form appears, as shown in Figure 9a. The CIR data were stored in I/Q format, and the phase values (unwrapped) of the FPI for each port were calculated using the 20 measured CIR data, shown as solid and dashed lines in Figure 9b. The phase information differences of each FPI are shown in the dotted line. The direction of the received signal can be estimated through a conventional PDoA method. Figure 10a shows the measured DoA according to the incident angle from −30° to 30° using ports 1 and 2 (*ϕ* = 0°). The DoA estimation results measured at distances of 30 cm and 60 cm are indicated by the blue and red solid lines, respectively. The results are compared with the ideal result denoted by the black solid line. The square marks represent the average measured values estimated by 20 signals, while the horizontal bars show the measurement error range with the maximum and minimum values, respectively. The root-mean-square (RMS) errors between the mean of the measured results and the ideal value are 1.57° at a distance of 30 cm and 4.58° at a distance of 60 cm. Figure 10b also shows the measured DoA estimation results using ports 1 and 3 (*ϕ* = 60°), and this measurement was conducted under the same condition. RMS errors of 2.48° and 4.72° are observed at distances of 30 cm and 60 cm, respectively. The results demonstrate that the proposed SRMP antenna using the tripod-shaped radiator is suitable for UWB direction-finding system usage in smart devices.

## 4. Conclusions

In this paper, we proposed an SRMP patch antenna for UWB direction-finding systems. Considering the limited mounting space of smart devices, the proposed antenna was designed using a single tripod-shaped patch that can replace the array system. The SRMP antenna was optimized using the edge shape design function of the tripod-shaped radiator to improve the broadband and mutual coupling characteristics. The proposed antenna had an operating frequency band of a bandwidth of 6.1 GHz for port 1 (from 5.8 GHz to 11.9 GHz). The simulation of −14.9 dB agreed well with the measurement of −13.3 dB at 8 GHz. In addition, the measured and simulated mutual couplings had similar results of −14.8 dB and −15.5 dB, respectively. The SRMP antenna had measured maximum gains of 3.5 dBi for port 1 and 2.9 dBi for port 2. To investigate the DoA estimation performance of the proposed antenna, the DW3000 chip, which is widely employed in commercial mobile UWB systems, was used. The proposed antenna was connected to the receiving module, and the CIR data were measured at 5° intervals at distances of 30 cm and 60 cm. The DoA results using the measured CIR data had RMS errors of 1.57° and 4.58°. The results demonstrated that the proposed SRMP antenna using a tripod-shaped radiator was suitable for UWB direction-finding system usage in small smart devices.

## Figures and Tables

**Table 1:** Optimized design parameters of the proposed SRMP antenna.

Parameter	Optimized Value
*w*_1_	10.1 mm
*w*_2_	4.87 mm
*l*_1_	11.7 mm
*r*_1_	15.05 mm
*r*_2_	12.8 mm
*h*	3.2 mm

**Table 2:** Comparison of the proposed SRMP antenna compared to the previous publications.

	Proposed Antenna	Previous Publication [29]	[26]	[27]
Aperture size	30.1 mm (0.8 λ at 8 GHz)	36 mm (0.96 λ at 8 GHz)	23.9 mm (5.1 λ at 64 GHz)	105 mm (1.05 λ at 3 GHz)
Operating frequency	5.8–11.9 GHz	7.03–10.0 GHz	57–71 GHz	3–17 GHz 25.3–35.1 GHz 35.5–49.4 GHz
Mutual coupling	less than −10 dB	less than −10 dB	less than −15 dB	less than −15 dB
Maximum gain	3.5 dBi	2.08 dBi	12.5 dBi	5.9 dBi
Performance verification using commercial system	Qorvo’s DW3000	-	-	-

## Data Availability

Data are contained within the article.
